# Relative Binding Affinity Prediction of Charge-Changing Sequence Mutations with FEP in Protein–Protein Interfaces

**DOI:** 10.1016/j.jmb.2019.02.003

**Published:** 2019-03-29

**Authors:** Anthony J. Clark, Christopher Negron, Kevin Hauser, Mengzhen Sun, Lingle Wang, Robert Abel, Richard A. Friesner

**Affiliations:** 1 Schrodinger Inc., 120 W 45th Street, New York, NY 10036, USA; 2 Department of Chemistry, Columbia University, 3000 Broadway, MC 3178, New York, NY 10027, USA

**Keywords:** free energy perturbation, antibodies, protein-protein binding, mm-GB/SA, molecular mechanics generalized Born surface area, MM, molecular mechanics, FEP, free energy perturbation, MD, molecular dynamics, bNAb, broadly neutralizing antibody, RMSE, root mean square error, SKEMPI, Structural database of Kinetics and Energetics of Mutant Protein Interactions, fSASA, fractional solvent accessible surface area

## Abstract

Building on the substantial progress that has been made in using free energy perturbation (FEP) methods to predict the relative binding affinities of small molecule ligands to proteins, we have previously shown that results of similar quality can be obtained in predicting the effect of mutations on the binding affinity of protein–protein complexes. However, these results were restricted to mutations which did not change the net charge of the side chains due to known difficulties with modeling perturbations involving a change in charge in FEP. Various methods have been proposed to address this problem. Here we apply the co-alchemical water approach to study the efficacy of FEP calculations of charge changing mutations at the protein–protein interface for the antibody–gp120 system investigated previously and three additional complexes. We achieve an overall root mean square error of 1.2 kcal/mol on a set of 106 cases involving a change in net charge selected by a simple suitability filter using side-chain predictions and solvent accessible surface area to be relevant to a biologic optimization project. Reasonable, although less precise, results are also obtained for the 44 more challenging mutations that involve buried residues, which may in some cases require substantial reorganization of the local protein structure, which can extend beyond the scope of a typical FEP simulation. We believe that the proposed prediction protocol will be of sufficient efficiency and accuracy to guide protein engineering projects for which optimization and/or maintenance of a high degree of binding affinity is a key objective.

## Graphical Abstract

**Figure:**
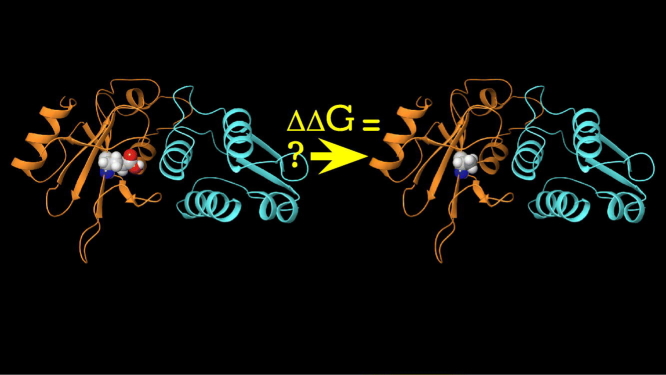

## Introduction

The prediction of the impact of residue mutations on protein–protein binding affinities is a major challenge for biomolecular simulation methodology. Protein–protein binding plays a critical role in a wide variety of biological processes, including antibody–antigen binding [1], gamma protein coupled receptor signaling [2], assembly of key molecular machines [3], and cell–cell recognition events (e.g., as mediated by cadherins) [4]. Computational assessment of binding affinity as a function of mutation would enable the specificity of these processes to be understood at an atomic level of detail. Furthermore, a robust and sufficiently accurate methodology could have a significant impact on the design of pharmaceutically useful biologics, such as monoclonal antibodies and vaccines.

A number of approaches have been taken for prediction of relative protein–protein binding affinities. Tools such as FoldX use empirically trained energy functions based on experimentally measured protein and protein complex stability data [5]. Methods such as molecular mechanics generalized Born surface area (mm-GB/SA) and molecular mechanics (MM) Poisson–Boltzmann surface area use MM models with implicit (continuum) solvent molecular models to provide a more physics based approach at somewhat more computational cost [6]. Other semi-empirical approaches have been developed that combine MM methods and additional energy terms optimized from experimental data [7]. Examples of available packages of this type include MutaBind [8], which combines terms from implicit solvent MM models with empirical energy functions and machine learning to train to experimental data, and BeatMusic, which is a statistics-based energy function derived from solved protein structures [9]. Free energy perturbation (FEP) is a fully physics-based model that uses explicitly represented water, with a series of separate molecular dynamics (MD) simulations (“lambda windows”) over which the weighting of the energy of a mutating residue is varied through intermediate alchemical states between wild and mutant type, where the free energy differences between each adjacent lambda window are calculated using a perturbative expansion and are summed to estimate the total free energy change [10]. In recent years, modern implementations have become valuable tools in small-molecule drug discovery projects [11], [12].

In a recent publication, we have carried out a large-scale test of the ability of (FEP) methodology to predict the change in binding free energy upon mutation for a series of mutants in antibodies binding to gp120, the viral spike protein of HIV-1 [11]. Optimization of the binding affinity of antibodies to a wide range of gp120 proteins, resulting in a broadly neutralizing antibody (bNAb) of high potency, is a major objective in developing antibody therapeutics as an alternative to small molecule treatment of HIV infection. Carrying out physically realistic FEP calculations required building homology models for a number of antibody–gp120 complexes and incorporating the effects of surface glycans upon antibody binding into the calculations. Despite these challenges, a root mean square error (RMSE) of 0.84 kcal/mol in comparison with experiment was achieved across a data set of 55 mutations, demonstrating that, with minor modifications of the sampling protocol, the FEP + methodology that we developed previously for small-molecule binding affinity calculations can successfully predict the effects of protein mutations upon protein–protein binding affinity to near chemical accuracy.

However, the test set examined in Ref. [11] consisted exclusively of mutations that did not change the net charge on the system; that is, charge changing mutations such as Ala to Glu were excluded from the data set. Although some attempts at applying explicit solvent free energy calculations to problems such as protein folding exist [13], we deliberately set aside mutations in the latter category due to the well-known technical difficulties in performing alchemical simulations in which the net charge on the system is altered [14], [15], [16], [17], [18], [19]. We apply a variant of the co-alchemical water FEP approach first proposed by Wallace and Shen [15] and Chen *et al*. [16]. The methodology is briefly summarized below; a more detailed description along with a comparison with other available methodologies for charge correction can be found in Ref. [20], which is focused on small-molecule/protein FEP calculations.

We have applied the alchemical ion FEP approach to a set of point mutations involving a normally charged residue (aspartate, glutamate, arginine, or lysine) and a residue normally neutral under physiological conditions for the antibody/gp120 system studied previously. We exclude cases involving proline, as its nonstandard backbone causes additional technical challenges, and cases where the mutant type side chain is HIS, as determination of the correct protonation state is particularly challenging without crystallographic evidence. We also include test cases from a number of additional interacting protein–protein complexes, using data taken from the Structural database of Kinetics and Energetics of Mutant Protein Interactions (SKEMPI) [21], in order to increase the size of the data set to a size from which preliminary statistical conclusions can be drawn. A total of 162 point mutations are considered in all, including 19 from the binding of VRC class antibodies to gp-120.

Small-molecule FEP calculations are generally restricted to predictions for which a significant change in protein conformation (e.g., DFG-in to DFG-out loop motion in a kinase) is not expected. Such motions are unlikely to take place on a typical FEP simulation timescale. In the case of charge changing residue mutations, substantial protein rearrangement can readily be induced if the mutation is performed on a residue that is buried in an environment that is inhospitable to the new target residue. Such situations would in many cases require much longer simulations to yield fully converged results; fortunately, they are also typically not of interest in a project aimed at optimizing binding affinity, as the great majority of such mutations will make binding more unfavorable. In what follows, we present a simple approach to classifying buried residues and show that for the remaining cases, the RMSE of the FEP calculations is in a satisfactory range (~ 1.2 kcal/mol). First, an implicit solvent side-chain re-prediction is used to eliminate 12 cases where a reasonable side-chain conformation cannot be achieved in the wild-type input structure. Second, mutations to residues of different expected charge state from buried wild-type residues are classified by their fractional solvent accessible surface area (fSASA), SASA normalized to the maximum SASA in a tripeptide configuration [22]. Using a cutoff of 10% fSASA, below which residue side chains are considered fully buried, does not eliminate any significantly favorable mutations experimentally and leaves 106 cases, which are at least partially solvent exposed.

The paper is organized as follows. The Data Sets section discusses the various data sets used to evaluate the FEP calculations. In the Results and Discussion section, results of FEP simulations are presented for all of the test cases and analyzed via comparison with experiment. We use an mm-GB/SA protocol that samples side-chain conformations in the input structure [14] to provide a comparison with a simpler (and computationally less expensive) approach, and discuss the results obtained with the empirical foldX method [5]; this enables us to ask whether FEP is adding value commensurate with the substantial computational cost. In the Models and Methods section, we discuss the computational methodology used in the paper, including the co-alchemical water approach, characterization of poorly fitting and buried residues, and some details of the FEP simulation methodology including the use of extended sampling to handle cases in which hydrogen bonds and/or salt bridges need to be broken or formed. Finally, in the Conclusion section, we summarize our results and discuss future directions.

## Data Sets

### HIV gp120/antibody data set

Some infected patients develop bNAbs against human immunodeficiency virus [1], [24], [25], [26]. These antibodies are not suitable themselves as therapeutics, but they provide a potential starting point for developing more potent antibodies and eventually for gaining insight into the evolutionary development of bNAbs against HIV-1, which could be used toward vaccine development. Reliable computational tools to predict affinity changes under protein residue sequence modifications could be of great use in driving the design of more potent antibodies, and being able to predict the effect of mutations between charged and neutral side chains is an essential part of such tools.

In Ref. [11], we reported experimental binding affinity measurements, for both neutral and charge changing mutations, for three VRC01 class antibodies: VRC01, VRC03, and VRC-PG04. A description of the experimental protocols and results, utilizing surface plasmon resonance measurements, can be found in Ref. [11], with the experimental free energy values for all of these mutations in the Supplementary Material (Table S1).

The structures of the antibody/gp120 complexes on which the experimental binding affinity measurements were made are not available in the Protein Data Bank. However, related structures, with variants of the gp120 sequence, have been crystallized. In Ref. [11], we used these structures as templates to build homology models. The details of the homology model building protocols, the sequence alignments used to build the homology models, and results of MD simulations to validate the stability of the models are reported in Ref. [11]. We also built glycan fragments as necessary based on the template structures, an essential task because the glycans can have a very significant effect on mutational binding free energy changes if they are in close proximity to the residue in question, and these are retained here for all cases considered.

### Additional test cases drawn from the SKEMPI database

There are fewer charged than neutral residues in the interface of the VRC01–class bNAbs with gp120; only 19 total cases of potentially charge changing mutations are contained in the alanine scanning set reported previously [11]. To build a more extensive validation data set for testing the FEP charge changing methodology, we consider also protein residue mutation examples from three protein–protein complexes taken from the public SKEMPI database [9]. For the purposes of selecting cases for analysis, a putative charge changing mutation is taken to be any mutation where one end state is one of the 4-amino-acid side chains (ASP, GLU, LYS, and ARG), which are generally expected to be charged at physiological pH, and any other amino acid side chain not in this set.

In selecting cases for FEP consideration, we aim to assemble a set of point mutations, most of which represent the type of mutations we hope to pursue that are most similar to that which might be pursued in a practical biologic optimization project, and we select systems where the types of structural issues in the gp120 study (need for homology models, important glycosylation) are not present to give the best test of the specific FEP charge correction methodology being tested. In an analogy to the successful application of FEP to small-molecule drug discovery projects [27], [28], we assume that the FEP calculations will be used prospectively to either increase affinity or maintain the maximum possible affinity while other properties are optimized, so predictions of mutations that cause favorable or no change in binding affinity will be the only ones made experimentally. Therefore, we require each system chosen to have at least one unambiguously favorable mutation (ΔΔG ≤ -0.5 kcal/mol), and where there are multiple similar systems that could be used, the one with the most experimentally favorable mutations is chosen. Second, a real optimization project seeking favorable or neutral mutations will likely not be dominated by mutations to alanine, as the data set is, so we require that each system chosen be at least 25% mutations to amino acid side chains other than alanine, Finally, since we have the opportunity with the larger data set, we eliminate systems with complicating issues (e.g., missing loops, glycosylation, small molecules bound) in order to best isolate the effects of the new FEP protocol from these other challenges. The systems with 15 or more putative charge changing mutations that were considered for inclusion are summarized in the SI in Table S1.

The systems chosen are the complex of barnase with barstar (PDB ID 1BRS), turkey ovomucoid third domain (OMTKY3) with subtilisin Carlsberg (PDB ID 1R0R), and OMTKY3 with *Streptomyces griseus* proteinase B (SGPB) (PDB ID 3SGB). We exclude from these only mutations where the mutant amino acid side chain does not physically fit into the reference wild-type structure (see Models and Methods for more details). The remaining set is split into 106 solvent accessible mutations and 44 buried mutations by fSASA, only the former of which we claim would be likely to be of practical interest in optimizing binding affinity of the complex.

### Overview of the final data set

The resulting experimental data set is summarized in Table 1. In total, it includes 150 point mutations for which the mutant side chain can be reasonably placed in the wild-type crystal/model structure. The dynamic range of affinity changes measured is very large and includes mutations measured to strongly stabilize binding (down to − 2.55 kcal/mol in the OMTKY–/SGPB complex) to those that strongly destabilize binding (up to 7.66 kcal/mol in the very tight binding barnase–barstar complex).

**Table 1:** Full data set: summary of the protein–protein complexes used, the number of experimental mutations contained in each, and the range of experimental ΔΔ*G* values (Min:Max) in kcal/mol

Protein 1	Protein 2	No. of mutations	Non-buried	Min:Max
VRC01	gp120-RSC3	6	5	− 0.27:1.63
VRC03	gp120-RSC3	8	6	− 0.38:2.65
VRCPG-04	gp120	5	4	− 0.74:2.69
Barnase	Barstar	15	4	− 0.89:7.66
Subtilisin Carlsberg	Turkey ovomucoid third domain	59	36	− 1.09:5.69
*S. griseus* proteinase B	Turkey ovomucoid third domain	57	50	− 2.55:5.90
Overall	–	150	106	− 2.55:7.66

## Results and Discussion

**Fig. 1:**
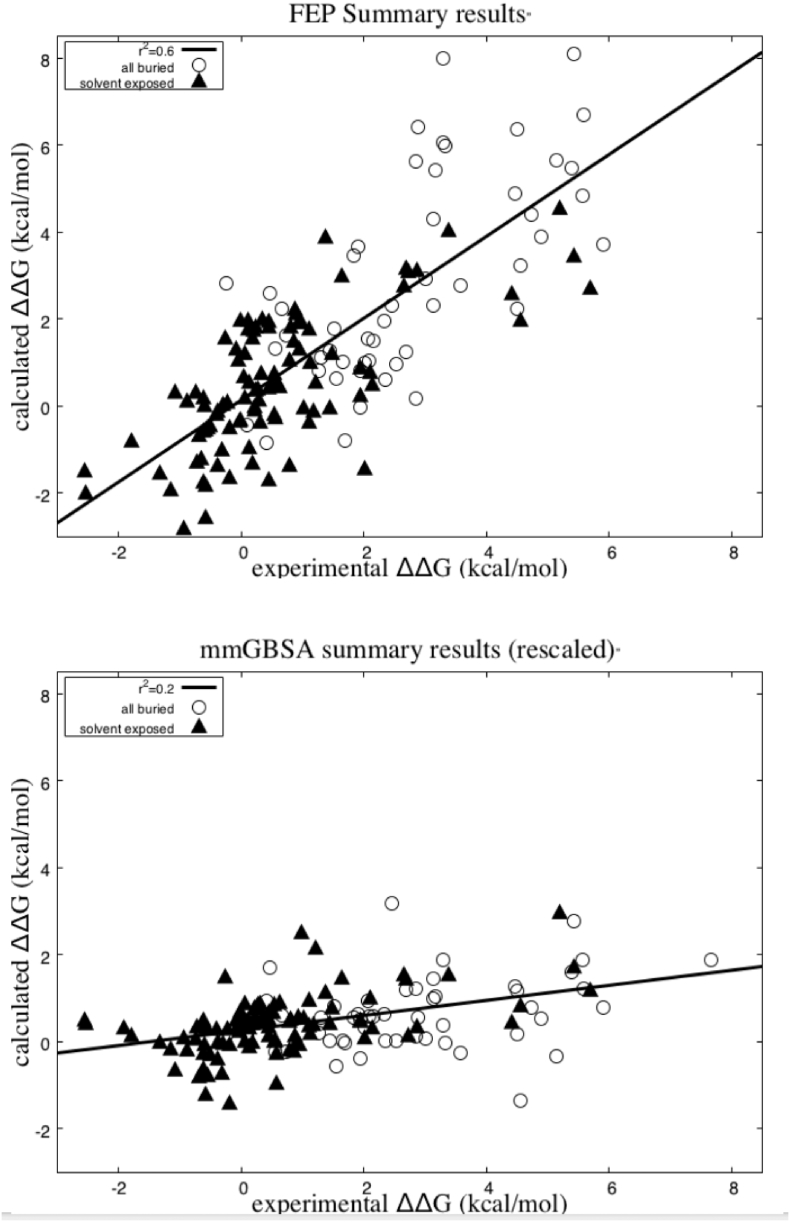
Summary results using FEP and the single-point mm-GB/SA protocol described in Models and Methods are shown. Coefficients of determination are given for all cases buried and unburied.

Using the protocol outlined in the Models and Methods section, we obtain estimates for the relative change in binding free energy from each of the set of 150 point mutations via FEP simulation. The results are summarized in Fig. 1, and RMSEs and coefficients of determination are given in Table 2. Figure 1 and Table 1 also provide the results of mm-GB/SA calculations for comparison. Figure 3 shows the location and wild-type charge of all positions where mutations were considered. In order for FEP to be a useful methodology, it must substantially outperform fast approximate methods like mm-GB/SA (or empirical alternatives such as Fold-X [29], with which comparisons were made in Ref. [11]) with regard to prediction accuracy.

**Table 2:** Summary of performance metrics for FEP and mm-GB/SA

Category	Methodology	RMSE (kcal/mol)	*R*^2^	*p*
Non-buried	FEP	1.23 [1.07–1.38] (1.22)	0.50 [0.36–0.62] (0.53)	< 0.001
Non-buried	mm-GB/SA	1.50 [1.22–1.76] (1.50)	0.18 [0.06–0.33] (0.23)	< 0.001
Buried	FEP	1.79 [1.41–2.11] (1.95)	0.52 [0.31–0.71] (0.40)	< 0.001
Buried	mm-GB/SA	2.80 [2.33–3.28] (2.44)	0.14 [0.0–0.41] (0.05)	0.014
All	FEP	1.41 [1.26–1.59] (1.40)	0.61 [0.5–0.71] (0.58)	< 0.001
All	mm-GB/SA	1.97 [1.71–2.21] (1.74)	0.17 [0.06–0.31] (0.23)	< 0.001

Error ranges for metrics (square brackets) are estimated based on a bootstrapping analysis using a 95% confidence interval. Values in parentheses show the result if the cases where acids are modeled entirely or partially, as protonated are excluded.

As anticipated, the “buried” residue mutations contain no cases significantly favorable to binding, and all but four decrease binding affinity significantly (by more than 0.5 kcal/mol); the magnitude of the free energy change is predicted with a coefficient of determination of 0.52 by FEP. The RMSE of these cases, however, is large, 1.79 kcal/mol. We believe that this is due to the inability to simulate significant conformational changes of the protein or protein–protein binding mode in the relatively short FEP trajectories. However, as was suggested above, it is not important in a practical project where the aim is to produce stronger binding proteins to determine whether a mutation will induce a free energy change of + 2 or + 5 kcal/mol; the mutation is not worth testing experimentally regardless of which is correct. Therefore, simply classifying a proposed mutation site as buried appears (at least based on this data set) to be sufficient to rule out a charge changing mutation at that site. We note that mm-GB/SA performs very poorly for test cases in this regime, with a coefficient of determination less than 0.1 and a very large RMSE (2.8 kcal/mol).

For the “solvent exposed” sites, FEP displays a much more reasonable RMSE of 1.22 kcal/mol, and a correlation of 0.53. mm-GB/SA also shows apparent improvement as compared to its performance on the buried residues. However, the improvement in the coefficient of determination is almost entirely due to some ability to discriminate highly unfavorable mutations from the remainder of the data set (a much easier problem than rank ordering favorable or nearly favorable mutations). To illustrate this observation, we present in Table 3 the coefficients of determination for the unburied data set with the relative experimental binding affinity truncated beyond + 1 kcal/mol. For this data set, FEP produces a coefficient of determination of 0.39, while mm-GB/SA displays close to zero correlation. These results imply that mm-GB/SA (and likely other related methods) would be useful in practice only as crude filters to eliminate very poorly scoring mutations, whereas FEP will preferentially generate a highly enriched set of candidates, including those leading to significant improvement in binding affinity.

**Table 3:** Correlation of FEP and mm-GB/SA predictions with experimental binding affinity changes < 1 kcal/mol

80 cases ΔΔ*G*_exp < 1	*R*^2^	*p*
FEP	0.39	< 0.001
mm-GB/SA	0.06	0.016

We have additionally compared the results to those found using foldX [5] and found the overall results to be very similar to those given by mm-GB/SA. FoldX performs similarly to mm-GB/SA, giving RMSEs of 1.8 and 1.5 kcal/mol for all mutations, and only non-buried mutations respectively, *versus* mm-GB/SA values of 1.9 and 1.5 kcal/mol. Furthermore, on cases with experimental value < 1 kcal/mol, FoldX gives a coefficient of determination of < 0.01 (*p* = 0.46). A plot is provided in the SI (Fig. S1).

**Fig. 2:**
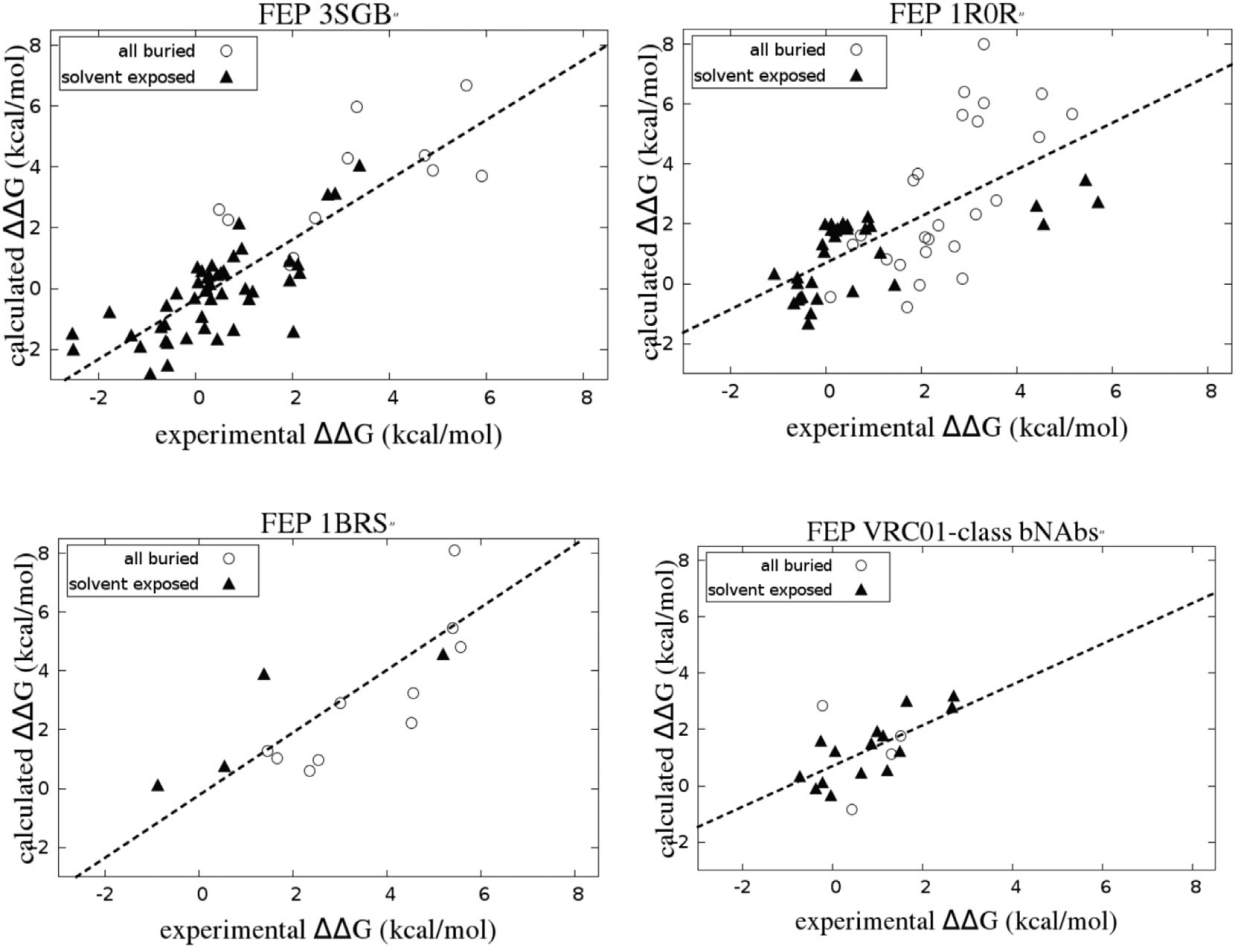
FEP results by system considered; results for the three VRC-01 class antibodies are combined.

Results for the various individual systems are shown in Fig. 2.

**Fig. 3:**
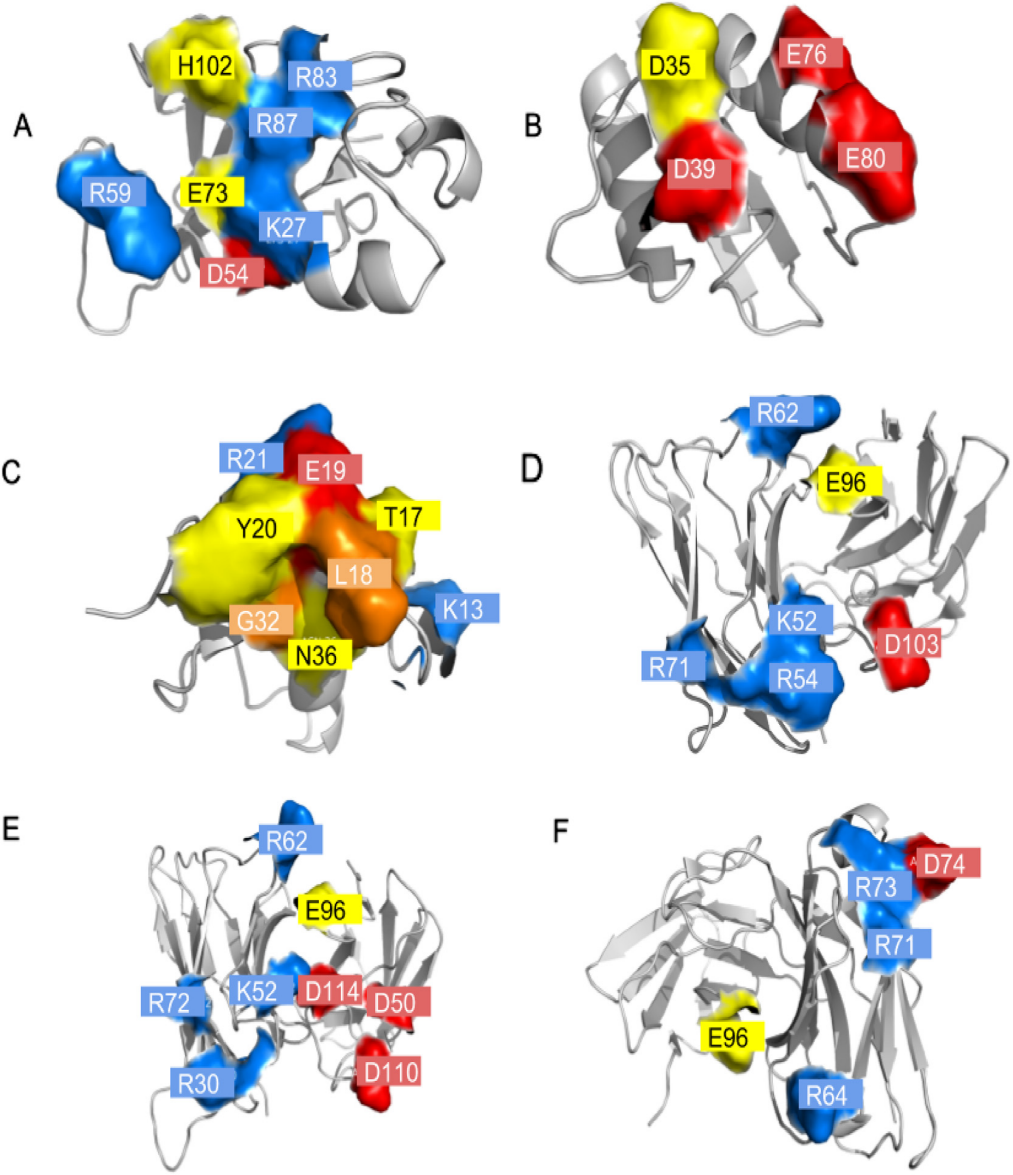
Surface map of protein binding sites showing the location of mutation sites for (A) barnase (B), barstar (C), OMTKY3, (D) VRC01, (E) VRC03, and (F) VRCPG-04.

### Challenges of FEP modeling of mutations at totally buried sites

When considering the effect on binding of a point mutation to a buried side chain with a different preferred charge state under neutral conditions, one of two scenarios is likely if the complex still binds at all: (1) the conformation of the complex changes to accommodate bulk solvent into the interface or (2) the protonation state of side-chain changes. An example of this is provided by the mutation of ASP39 on barstar, which disrupts a very stable salt-bridge network. Despite the complex binding with around 7 kcal/mol less affinity than the wild type, this mutant has been crystallized (PDB ID 2ZA4) [19], and the result shows a subtle change to the protein–protein binding mode that allows a column of water to penetrate the interface and solvate the residues that engage in the salt-bridge network in the wild-type structure. In this case, we under-predict the binding of the mutant complex in FEP, but the simulation is clearly not converged, with the relative binding free energy estimate still trending toward the experimental value after 100 ns.

In at least some cases, the complex may actually form in the same conformation, but with a neutral state of the relevant acid side chain. This is particularly plausible for protonated forms of carboxylate side chains (ASP and GLU), which are geometrically smaller than the basic side chains (ARG and LYS) and so are more likely to be able to fit without steric clashes in the space occupied by the surrounding protein structure in the wild-type configuration. Indeed, the test set contains a number of buried wild-type acid residues [see the summary results table in the supporting information (SI)]. In such cases, we model both states explicitly in FEP, incorporating an approximate state penalty in the case that the side chain is believed to be deprotonated in the unbound state and protonates on binding, and the state with the lower implied free energy of binding is used for comparison with experiment. As described in Ref. [16], a p*K*_a_ correction must be applied in cases where the state changes between the unbound and bound state. Due to the extreme challenges of accurately calculating the p*K*_a_ shift experienced by a buried residue due to the local protein environment, we have approximated the correction by assuming that the p*K*_a_ shift is very large and the population in the bound state is entirely protonated. Table 2 includes values for all metrics without these cases in the data set; in all cases, the RMSE and coefficient of determination are within the uncertainty range of the full set with these cases included, and so their inclusion does not affect our overall conclusions. All cases where a mutant acid side chain is believed to be protonated are very unfavorable to binding, but several cases with wild-type acid side chains that are believed to protonated would result in false-positive predictions of binding affinity improvement if naively modeled as charged, making incorporating this analysis of buried residues important for performance in an optimization effort. All cases where non-standard protonation states are used are listed in Table 4.

**Table 4:** Table of nonstandard protonation states for carboxylate side chains

PDB ID/template PDB ID	Chain	Position	WT residue code	MT residue code	WT fSASA (%)	Complex state	Unbound state	Comments	FEP result (kcal/mol)	FEP if modeled as charged (kcal/mol)
1BRS (barnase–barstar)	A	73	E	Q	1	Wild type: GLH	Wild type: GLH	Hydrogen bond with ASP75 on same chain	1.27	− 8.45
1BRS	A	73	E	C	1	Wild type: GLH	Wild type: GLH	Hydrogen bond with ASP75 on same chain	0.96	− 3.23
1BRS	A	73	E	S	1	Wild type: GLH	Wild type: GLH	Hydrogen bond with ASP75 on same chain	2.92	− 2.86
1BRS	A	73	E	A	1	Wild type: GLH	Wild type: GLH	Hydrogen bond with ASP75 on same chain	0.60	− 10.02
1BRS	A	102	H	D	0	Mutant type: ASH	Mutant type: ASH	Mutant in close proximity to ASP39 on same chain; stabilizing hydrogen bond	3.23	28.89
1BRS	D	35	D	A	1	Wild type: ASH	Wild type: ASH	Fully buried	2.24	0.73
1R0R (OMTKY3–subtilisin Carlsberg)	I	15	A	D	0	Mutant type: ASH	Mutant type: ASP	Buried in mostly hydrophobic pocket of receptor in bound state	5.66	18.03
1R0R	I	15	A	E	0	Mutant type: GLH	Mutant type: GLU	Buried in mostly hydrophobic pocket of receptor in bound state	6.35	13.34
1R0R	I	18	L	D	5	Mutant type: ASH	Mutant type: ASP	Buried in mostly hydrophobic pocket of receptor in bound state	4.89	11.36
1R0R	I	18	L	E	5	Mutant type: GLH	Mutant type: GLU	Buried in mostly hydrophobic pocket of receptor in bound state	1.49	5.58
3SGB (OMTKY–SGPB)	I	17	T	D	12	Mutant type: ASH	Mutant type: ASP	Buried in mostly hydrophobic pocket of receptor in bound state	3.88	7.81
3SGB	I	17	T	E	12	Mutant type: GLH	Mutant type: GLU	Buried in mostly hydrophobic pocket of receptor in bound state	4.39	5.38
3SGB	I	18	L	D	1	Mutant type: ASH	Mutant type: ASP	Buried in mostly hydrophobic pocket of receptor in bound state	6.70	15.13
3SGB	I	18	L	E	1	Mutant type: GLH	Mutant type: GLU	Buried in mostly hydrophobic pocket of receptor in bound state	3.70	11.4
VRC01 [3NGB Template]	L	96	E	A	2	Wild type: GLH	Wild type: GLH	Caged by aromatics on antibody light chain	1.11	− 0.49
VRC03 [3SE8 template]	L	96	E	A	1	Wild type: GLH	Wild type: GLH	Caged by aromatics on antibody light chain	1.77	− 1.13
VRCPG-04 [3SE9 template]	L	96	E	A	3	Wild type: GLH	Wild type: GLH	Caged by aromatics on antibody light chain	− 0.84	− 0.74

GLH and ASH refer to the protonated form of the carboxylate in glutamic and aspartic acid respectively, while GLU and ASP refer to the deprotonated (charged) forms of the same.

**Fig. 4:**
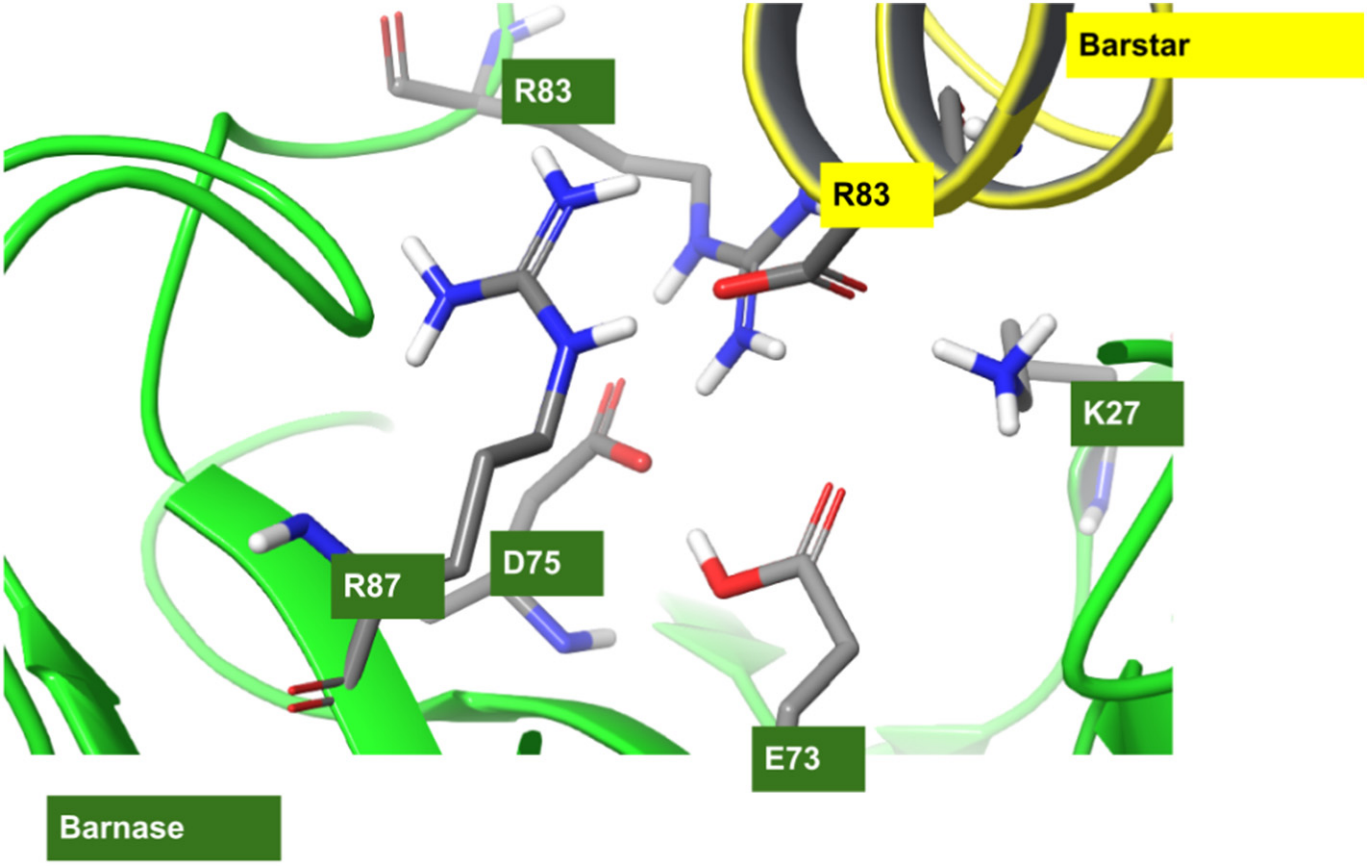
The protonated GLU-73 side chain (bottom) is observed to form stabilizing hydrogen bond with deprotonated ASP-75 side chain to stabilize a key buried salt-bridge network in the complex of barnase and barstar.

One scenario where the protonated form of a buried acid residue may be particularly favored is when it is in close proximity to another acid side chain and a hydrogen bond network involving the protonated carboxylate forms with the neighboring group. A well-studied canonical example of this scenario is the aspartate proteases, particularly HIV protease, a protein homodimer where one of the two proximal catalytic aspartates is believed to be protonated [30], [31], [32], [33]. The prominent example of this effect we believe is occurring in the data set here is glutamic acid at position 73 on wild-type barnase, where the protonated form is found in FEP trajectories to form a stabilizing hydrogen bond with an adjacent carboxylate side chain (ASP-75 or ASP-39, see Fig. 4). Previous experimental work using double mutant cycles found a strong implied coupling between this residue and ASP39, which they describe as “…surprising, since Glu73 does not interact directly with barstar and there is an electrostatic repulsion between Glu73 on barnase and the negatively charged binding surface of barstar” [24]. This configuration is not found in the crystal structure model 1BRS, but reliably forms and persists in MD. FEP calculations for the mutation using both the charged and neutral forms of the GLU side chain and perturbing to the same neutral side chain suggest that the neutral form is preferred by a significant factor. Starting from the charged form, FEP predicts an increase in the binding affinity of the mutant compared to the wild-type complex by > 2 kcal/mol for all cases. Starting from the neutral form, FEP recovers the correct classification of all these mutants (unfavorable). Asp75, also on barnase, is believed to be partially responsible for the effect, Glu73 is also modeled as neutral in the unbound form, and no state penalty is incurred upon binding.

While these specific cases are not likely of relevance for biologic affinity optimization, the use of FEP simulations to assess the protonation states of ionizable residues (as discussed above) may have potential applications. Assignment of protonation states in buried parts of protein interfaces is a very difficult problem, and this approach could be used in combination with a small amount of experimental mutagenesis data to determine the likely preferred protonation state assignment.

## Models and Methods

### FEP protocol for charge perturbations

Accurate calculation of the change in the binding free energy for mutations that change the net charge of the residues is difficult for multiple reasons. First, the great majority of MD simulations employ periodic boundary condition for approximating the behavior of macro-system by using a finite sized simulation box, and the periodic boundary condition introduces artifacts for electrostatic potential energy calculations for charged solutes. The artifacts can be decomposed into the net charge interaction between the solute and its periodic images, the under-solvation of the solute due to the finite size of the simulation box, and the inconsistency of zero electrostatic potential in simulations of different systems [14]. Second, the experimental binding affinity assays are usually conducted in a buffer solution with salts, and the binding affinity between the charged solutes are critically dependent on the ionic strength of the buffer solution, which is very difficult to model accurately. Third, mutations changing the net charge of the residue often have very different interactions with their protein binding partner and the surrounding water molecules, and can be very difficult to converge free energy calculations when these differential interactions lead to substantial conformational changes.

In this paper, we have tested a variant of the co-alchemical particle approach first introduced in Refs. [15], [16] and studied extensively for small molecules in Ref. [16] to calculate the effect of charge changing residue mutation to the protein–protein binding free energy. The protocol used is the same as described in Ref. [11], using FEP + with the OPLS3 force field, except for the following modifications to adapt to the co-alchemical water approach:

(1) The charge conserving protocol through the introduction of a co-alchemical particle. In particular, for a mutation from a neutral residue to a charged residue, a particle is co-mutated with the residue mutation, from a neutral particle into a sodium ion (mutated residue has − 1 charge) or chloride ion (mutated residue has + 1 charge). The ion is chosen randomly from the ions placed in the box and mutates to a neutral particle. No additional restraints are put on the ion to prevent it from entering the protein. In this way, the net charge change between the two physical end states is zero, and the simulation box size-dependent artifacts in the electrostatic potential energy calculations are eliminated to within the uncertainty of the free energy calculation [20], which we estimate based on repeated trials of the same perturbation with different randomized initial velocity conditions to be approximately 0.5 kcal/mol [11].
(2) To model the effect of the ionic strength in the buffer solution on the protein–protein binding free energy, sodium and chloride ions with concentrations matching the ionic strength of the assay condition are explicitly added in the simulations. These ions are placed by randomly replacing waters in the initial solvent buffer generated.
(3) To enhance the convergence of the free energy calculations, the number of lambda windows in FEP + simulations is increased from 12 for charge conserving mutations to 24 for the charge changing residue mutations, with the weights for the electrostatic term of the energy further optimized in order to maximizing the configurational space overlap between neighboring lambda windows (see the SI section Lambda Weights for this non-uniform schedule of weights). In addition, a larger solvent buffer, 8.5 Å rather than 5 Å, is used for the simulation box. Together, these modifications have been found to consistently produce good energy overlap between adjacent lambda windows and good convergence with increasing simulation time for a large number of cases. Furthermore, perturbations involving residues in multipart salt bridges in the wild type are run with much longer simulation time (100 ns per window), as well as the residues on the glycosylated gp120 antibodies, where a glycan fragment is retained in the simulated system [11].

Through these technological improvements, the difficulties preventing the accurate modeling of charge charging residue mutations are effectively addressed.

### Assessment of the suitability of charge changing mutation test cases for FEP

Two of the data sets used herein are examples of systematic scanning of all possible amino acid residues at each position in the binding interface that is considered. In this approach, a subset of attempted mutations will involve placing side chains into positions where significant structure rearrangements will likely be necessary in order for the proteins to bind. Such cases will generally be highly destabilizing to the binding of the complex, and finding a reasonable starting geometry to use for the alchemical FEP simulation may not be possible. Furthermore, in such cases, it is very unlikely that FEP will be able to sample sufficiently to predict the correct change in binding affinity.

To identify cases where a reasonable side-chain placement is not possible, we performed implicit solvent side-chain predictions for the mutant residue in the wild-type structure and any potentially clashing neighbor side chains. The internal energy of the side chain conformation predicted is calculated using the OPLS3 force field and compared to the same energy of an optimized rotamer from a rotamer library. If the resulting intra-side chain strain energy in the system (deviation from the energy of the optimized rotamer) exceeded a 5-kcal/mol increase from the wild-type system, it was concluded that a reasonable starting geometry for the side chain was not possible and the point mutation was not taken forward for FEP. This threshold is chosen to as a fixed value, which is a significant fraction of the total binding affinity of the systems considered here (which are in the range of − 10 to − 20 kcal/mol). The exact threshold that should be used will likely require further optimization. This affected only 11 cases out of an initial 161 initially considered. The specific cases are noted in the summary results table in the SI.

### Categorization of mutations involving buried residues

For the remaining cases, fSASA was used to categorize the mutation site in the input structure as buried (fSASA < 10%; 44 total cases) and solvent exposed (fSASA ≥ 10%; 106 total cases). The definition of fSASA is taken to be the fraction of the maximum possible solvent accessible surface area in a tripeptide configuration experienced by the residue in the interface [13]. We note here that all mutations with a significant favorable effect on binding are contained in the latter set, and FEP performance is good (RMSE 1.23) on the set of non-buried positions. For the purposes of a practical optimization project where increased affinity is a goal, we therefore propose that fSASA can be used as a second easily automatable filter to determine which potentially charge changing mutations to send to FEP predictions. For scientific interest, we also examine here the 44 buried cases and attempt to understand their behavior in FEP.

### mm-GB/SA protocol

For this work, we used the mm-GB/SA approach implemented in BioLuminate (version 3.0.011, Schrödinger, LLC, New York, NY, 2018) to predict relative changes in binding affinity at protein–protein interfaces upon an amino acid mutation [34]. This method integrates the OPLS force field [35], with the implicit solvent model VSGB [36], and the rotamer library and search method of Prime [37]. Conformational sampling is limited to side chains only; the protein backbone remains fixed, and no MD sampling is used in this protocol. Input structures for all complexes and a spreadsheet of all FEP results are available as supplementary so that the reader may compare with mm-GB/SA methodologies with additional sampling employed. The integration of OPLS with the VSGB model combines the OPLS modeling of bonded and non-bonded terms with the solvation and desolvation energies of the VSGB model, which additionally contains physics-based correction terms. It should be noted that there is no term for changes in side-chain configurational entropy, but despite this limitation, the method has performed just as well as other mm-GB/SA implementations [38]. Crystallographic waters were deleted from the mm-GB/SA input structures as they degraded performance slightly (the RMSE is increased from 1.95 to 2.09 kcal/mol and reducing *r*^2^ from 0.22 to 0.13) for the systems explored in this study. Results including crystallographic waters can be found in the SI in Table S3.

Previous work by Beard *et al*. [34] observed that the optimal slope for converting Prime energies to experimental energies was system dependent. Thus, the rescaled Prime energies were determined in the following manner to get the optimal performance from Prime. The data set was divided into four subsets. Three of those subsets correspond to the three subsets of experimental data derived from the SKEMPI database (i.e., Barnase:Barstar, Subtilisin Carlsberg:OMTKY3, and *S. griseus* proteinase B:OMTKY3). The last subset of data corresponds to all experimental measurements involving VRC antibodies. The VRC antibody data were pulled together into a single subset in order to create a subset of data of reasonable size and spread in energy (19 mutations). The best fit line was then calculated for each subset of the data between the Prime predicted changes in relative binding affinity and the experimentally measured changes in relative binding affinity. The best-fit slopes ranged from 0.07 to 0.14. The slopes of those lines were used to rescale the Prime predicted energies for each subset. Those rescaled energies were then used to calculate the final RMSE and *r*^2^ values reported in this work.

### Sampling of salt-bridge networks

A relatively frequently occurring sampling challenge for FEP in charge changing mutations at a protein–protein interface is that the change in charge may disrupt salt-bridge networks that extend across several residues. Therefore, when mutations are made to residues identified as participating in multiple salt bridges (including bidentate motifs) where large energetic barriers to sampling out of the initial conformation are expected due to the strong electrostatic forces involved, simulation time is extended out to 100 ns. For the HIV bNAb cases, which use homology models where there is additional uncertainty in the placement of charged residues at the surface where they have been modeled in in place of neutral side chains, we extend all simulation times to 100 ns to allow more complete sampling. In addition, if a new salt bridge is observed to form in the simulation trajectory, the simulation is re-run with the mutant-type salt-bridge partner side chains in the REST region, to allow sampling out of the initially formed salt bridge.

## Conclusion

The FEP calculations shown here are relatively inexpensive, costing approximately $10–15 per simulation given a price point for GPU time around $0.40 per GPU hour, and most can be completed within ~ 8 h. Consequently, it is feasible to run thousands or tens of thousands of calculations in order to explore potential improvements to a biological therapeutic such as an antibody or vaccine. The issue of what kind of impact such calculations can have on a project, given the availability of methods like phage display to combinatorically explore sequence space experimentally, remains to be seen. The ability to target particular mutations of interest, and to at least estimate the effects on other properties which have to be optimized in order to create a development candidate, will be important in achieving significant impact in practical applications.

A second type of application of protein residue FEP would be to gain insight into selectivity of important biological recognition events such as cadherin binding. Minor sequence changes in cadherins are critical to different types of cells recognizing one another (and rejecting alternative cell types). In principle, FEP can provide an accurate atomic level basis for such recognition, given sufficiently good starting structures. Explorations along these lines using FEP have yet to be attempted at a large scale.

In conjunction with recently obtained results for small-molecule ligand binding, the present work provides solid evidence that the co-alchemical water approach is a satisfactory solution to the problem of applying FEP to transformations involving a change in net charge. The RMSE for charge changing mutations is somewhat larger than that for neutral mutations, even for solvent exposed sites. We suspect that this is due to the greater possibility of nontrivial protein reorganization, even if the site does not fall into the “buried” category as we have defined it here. Improved sampling protocols and simple brute force increases in simulation length should address these issues over time.

## Appendix A Supplementary data

Supplimentary text Image 1

Supplementary prepared structure, barnase-barstar Image 2

Supplementary prepared structure, OMTKY3-SGPB Image 3

Supplementary prepared structure, OMTKY3-Subtilsen Carlsberg Image 4

Supplementary prepared structure, VRC01-gp120(RSC3) homology model Image 5

Supplementary prepared structure, VRC03-gp120(RSC3) homology model Image 6

Supplementary prepared structure, VRCPG04-gp120(RSC3) homology model Image 7

Full spreadsheet of FEP data. Image 8
