# Supplementary material for: Relative Binding Affinity Prediction of Charge-Changing Sequence Mutations with FEP in Protein–Protein Interfaces
Source: J Mol Biol. 2019 Mar 29;431(7):1481–93. doi: 10.1016/j.jmb.2019.02.003 (PMC6453258; doi:10.1016/j.jmb.2019.02.003)
Supplement: Supplementary file 1 — Supplimentary text [file mmc1.doc]

**Supporting Information**

**Selection of Additional Validation data from SKEMPI Set**

| 1CHO | 66 | 63 | 2 | 95.45% | Less favorable mutations than other OMTKY3 systems; significant unresolved structure |
| --- | --- | --- | --- | --- | --- |
| 1PPF | 66 | 63 | 7 | 95.45% | less favorable mutations than other OMTKY3 systems, glycosylated |
| 1R0R | 66 | 63 | 8 | 95.45% | -- |
| 3SGB | 66 | 63 | 14 | 95.45% | -- |
| 1A22 | 52 | 2 | 1 | 3.85% | not enough non-alanine cases |
| 1DAN | 30 | 1 | 0 | 3.33% | not enough non-alanine cases |
| 1GC1 | 25 | 0 | 0 | 0.00% | out; not enough non-alanine |
| 3HFM | 24 | 18 | 0 | 75.00% | out; no favorable mutations |
| 1IAR | 19 | 13 | 1 | 68.42% | Missing loops needed for MD stability |
| 1BRS | 18 | 8 | 1 | 44.44% | -- |
| 1CBW | 17 | 12 | 5 | 70.59% | missing loops near protein-protein loops |
| 1LFD | 15 | 8 | 5 | 53.33% | ligand bound near interface |
| 1A4Y | 15 | 2 | 0 | 13.33% |  |

Table S1: Systems from SKEMPI with at least 15 putative charge-changing mutations

**Comparison with FoldX**

**
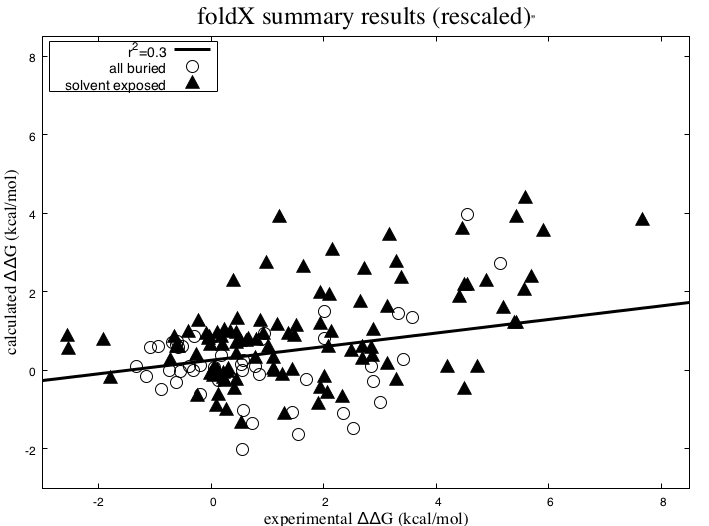
**

Figure S1: FoldX predictions vs experimentally measured change in binding affinity. With RMSE values of 2.45 kcal/mol for buried cases, 1.50 kcal/mol for non-buried cases, and 1.83 kcal/mol overall, FoldX gives nearly the same performance as mm-GB/SA

**Full results table**

| case | Experiment (kcal/mol) | Summary FEP (kcal/mol) | fractional SASA | Notes on protocol |
| --- | --- | --- | --- | --- |
| VRCPG-04R73 | -0.23 | 0.11 | 0.24 | -- |
| VRCPG-04R71 | 2.69 | 3.19 | 0.14 | -- |
| VRCPG-04R64 | 1.48 | 1.21 | 0.28 | -- |
| VRCPG-04D74 | -0.74 | 0.32 | 0.67 | -- |
| VRC03R72 | 2.65 | 2.78 | 0.15 | -- |
| VRC03R62 | 1.21 | 0.55 | 0.18 | -- |
| VRC03R30 | -0.04 | -0.34 | 0.17 | -- |
| VRC03K52 | 1.11 | 1.78 | 0.15 | -- |
| VRC03D49 | 0.86 | 1.49 | 0.21 | -- |
| VRC03D110 | -0.38 | -0.11 | 0.79 | -- |
| VRC01R71 | 1.64 | 3.01 | 0.11 | -- |
| VRC01R61 | 0.99 | 1.92 | 0.10 | -- |
| VRC01R53 | -0.27 | 1.58 | 0.31 | -- |
| VRC01K52 | 0.62 | 0.45 | 0.12 | -- |
| VRC01D99 | 0.05 | 1.23 | 0.56 | -- |
| 3SGB_I_Y20R | 2.72 | 3.10 | 0.14 | -- |
| 3SGB_I_Y20K | 3.38 | 4.05 | 0.14 | -- |
| 3SGB_I_Y20E | 2.01 | -1.44 | 0.14 | REST sampling expanded; observed salt bridge formation |
| 3SGB_I_Y20D | 2.87 | 3.12 | 0.14 | -- |
| 3SGB_I_T17R | 1.94 | -- | 0.12 | excluded; could not place mutant side chain |
| 3SGB_I_T17K | 2.01 | 1.00 | 0.12 | -- |
| 3SGB_I_T17E | 4.73 | 4.39 | 0.12 | -- |
| 3SGB_I_T17D | 4.89 | 3.88 | 0.12 | -- |
| 3SGB_I_R21Y | 0.29 | 0.14 | 0.46 | -- |
| 3SGB_I_R21W | 0.31 | -0.36 | 0.46 | -- |
| 3SGB_I_R21V | -0.02 | -0.31 | 0.46 | -- |
| 3SGB_I_R21T | 0.45 | 0.44 | 0.46 | -- |
| 3SGB_I_R21S | 0.27 | 0.41 | 0.46 | -- |
| 3SGB_I_R21Q | 0.04 | 0.68 | 0.46 | -- |
| 3SGB_I_R21N | 0.33 | -- | 0.46 | excluded; could not place mutant side chain |
| 3SGB_I_R21M | 0.13 | 0.56 | 0.46 | -- |
| 3SGB_I_R21L | 0.22 | -0.01 | 0.46 | -- |
| 3SGB_I_R21I | 0.24 | 0.37 | 0.46 | -- |
| 3SGB_I_R21H | 0.45 | -1.68 | 0.46 | -- |
| 3SGB_I_R21F | 0.22 | -0.09 | 0.46 | -- |
| 3SGB_I_R21C | 0.54 | -0.18 | 0.46 | -- |
| 3SGB_I_R21A | 0.05 | 0.19 | 0.46 | -- |
| 3SGB_I_N36R | 0.57 | 0.47 | 0.17 | -- |
| 3SGB_I_N36K | 0.57 | 0.57 | 0.17 | -- |
| 3SGB_I_N36E | 0.95 | 1.31 | 0.17 | -- |
| 3SGB_I_N36D | 0.90 | 2.14 | 0.17 | -- |
| 3SGB_I_K13Y | -0.58 | -- | 0.12 | excluded; could not place mutant side chain |
| 3SGB_I_K13W | -0.01 | -- | 0.12 | excluded; could not place mutant side chain |
| 3SGB_I_K13V | -0.94 | -2.80 | 0.12 | -- |
| 3SGB_I_K13T | -1.92 | -3.21 | 0.12 | -- |
| 3SGB_I_K13S | -2.55 | -1.49 | 0.12 | -- |
| 3SGB_I_K13Q | -0.61 | -0.57 | 0.12 | -- |
| 3SGB_I_K13N | -0.65 | -1.19 | 0.12 | -- |
| 3SGB_I_K13M | -1.15 | -1.92 | 0.12 | -- |
| 3SGB_I_K13L | -1.79 | -0.78 | 0.12 | -- |
| 3SGB_I_K13I | -1.33 | -1.54 | 0.12 | -- |
| 3SGB_I_K13H | -0.40 | -0.18 | 0.12 | -- |
| 3SGB_I_K13F | -0.72 | -1.28 | 0.12 | -- |
| 3SGB_I_K13C | -0.58 | -2.54 | 0.12 | -- |
| 3SGB_I_K13A | -2.54 | -2.00 | 0.12 | -- |
| 3SGB_EI19Y | 0.78 | 1.07 | 0.14 | -- |
| 3SGB_EI19V | 0.13 | -0.95 | 0.14 | -- |
| 3SGB_EI19T | 2.14 | 0.51 | 0.14 | -- |
| 3SGB_EI19S | 1.94 | 0.25 | 0.14 | -- |
| 3SGB_EI19Q | 0.18 | -1.31 | 0.14 | -- |
| 3SGB_EI19N | 1.10 | -0.36 | 0.14 | -- |
| 3SGB_EI19M | -0.19 | -1.63 | 0.14 | -- |
| 3SGB_EI19L | 0.78 | -1.36 | 0.14 | -- |
| 3SGB_EI19I | -0.62 | -1.72 | 0.14 | -- |
| 3SGB_EI19H | 0.52 | 0.50 | 0.14 | -- |
| 3SGB_EI19G | 2.10 | 0.79 | 0.14 | -- |
| 3SGB_EI19F | 1.94 | 0.90 | 0.14 | -- |
| 3SGB_EI19C | 1.17 | -0.10 | 0.14 | -- |
| 3SGB_EI19A | 1.02 | -0.02 | 0.14 | -- |
| 1R0R_I_Y20R | 4.41 | 2.60 | 0.12 | -- |
| 1R0R_I_Y20K | 5.43 | 3.45 | 0.12 | -- |
| 1R0R_I_Y20E | 4.55 | 1.98 | 0.12 | -- |
| 1R0R_I_Y20D | 5.69 | 2.71 | 0.12 | -- |
| 1R0R_I_R21Y | 0.81 | 1.83 | 0.49 | -- |
| 1R0R_I_R21W | 0.93 | 1.93 | 0.49 | -- |
| 1R0R_I_R21V | 0.11 | 1.79 | 0.49 | -- |
| 1R0R_I_R21T | 0.34 | 2.01 | 0.49 | -- |
| 1R0R_I_R21S | -0.02 | 1.97 | 0.49 | -- |
| 1R0R_I_R21Q | 0.18 | 1.58 | 0.49 | -- |
| 1R0R_I_R21N | -0.05 | 1.06 | 0.49 | -- |
| 1R0R_I_R21M | 0.44 | 1.82 | 0.49 | -- |
| 1R0R_I_R21L | 0.23 | 1.82 | 0.49 | -- |
| 1R0R_I_R21I | 0.20 | 1.76 | 0.49 | -- |
| 1R0R_I_R21F | 0.44 | 1.96 | 0.49 | -- |
| 1R0R_I_R21C | 0.11 | 1.98 | 0.49 | -- |
| 1R0R_I_R21A | -0.09 | 1.32 | 0.49 | -- |
| 1R0R_I_N36R | 1.44 | -0.03 | 0.22 | -- |
| 1R0R_I_N36K | 1.13 | 1.02 | 0.22 | -- |
| 1R0R_I_N36E | -0.20 | -0.49 | 0.22 | -- |
| 1R0R_I_N36D | 0.56 | -0.27 | 0.22 | -- |
| 1R0R_I_K13Y | -0.69 | -0.66 | 0.27 | -- |
| 1R0R_I_K13W | -1.09 | 0.33 | 0.27 | -- |
| 1R0R_I_K13V | 0.40 | 2.07 | 0.27 | -- |
| 1R0R_I_K13T | 0.88 | 2.24 | 0.27 | -- |
| 1R0R_I_K13S | -0.30 | 0.05 | 0.27 | -- |
| 1R0R_I_K13Q | -0.52 | -0.44 | 0.27 | -- |
| 1R0R_I_K13N | -0.39 | -- | 0.27 | excluded; could not place mutant side chain |
| 1R0R_I_K13M | -0.55 | -0.48 | 0.27 | -- |
| 1R0R_I_K13L | -0.32 | -1.00 | 0.27 | -- |
| 1R0R_I_K13H | -0.58 | -0.53 | 0.27 | -- |
| 1R0R_I_K13C | -0.61 | 0.21 | 0.27 | -- |
| 1R0R_I_K13A | -0.60 | 0.02 | 0.27 | -- |
| 1R0R_I_G32R | 3.42 | 2.05 | 0.10 | -- |
| 1R0R_I_G32K | 2.89 | 2.88 | 0.10 | -- |
| 1R0R_I_G32E | 1.95 | -0.03 | 0.10 | -- |
| 1R0R_I_G32D | 2.85 | 0.17 | 0.10 | -- |
| 1BRS_D_E80A | 0.54 | 0.77 | 0.63 | -- |
| 1BRS_D_E76A | 1.37 | 3.88 | 0.29 | -- |
| 1BRS_A_R59K | 2.49 | 0.32 | 0.19 | -- |
| 1BRS_A_R59A | 5.19 | 4.56 | 0.19 | -- |
| 1BRS_A_D54A | -0.89 | 0.12 | 0.14 | -- |

**Table S2: Summary data, “non-buried” cases**

| case | experiment | summary FEP | frac sasa | notes |
| --- | --- | --- | --- | --- |
| VRCPG-04_L_E96A | 0.42 | -0.84 | 0.03 | 100ns |
| VRC03_L_E91A | 1.51 | 1.77 | 0.01 | 100ns |
| VRC03_H_D114A | -0.24 | 2.83 | 0.09 | 100ns |
| VRC01_L_E96A | 1.31 | 1.11 | 0.02 | 100ns |
| 3SGB_I_L18R | 3.32 | 5.99 | 0.01 | -- |
| 3SGB_I_L18K | 3.14 | 4.29 | 0.01 | -- |
| 3SGB_I_L18E | 5.90 | 3.70 | 0.01 | -- |
| 3SGB_I_L18D | 5.59 | 6.70 | 0.01 | -- |
| 3SGB_I_G32R | 3.46 | -- | 0.08 | excluded; could not place mutant side chain |
| 3SGB_I_G32K | 2.49 | -- | 0.08 | excluded; could not place mutant side chain |
| 3SGB_I_G32E | 1.94 | -- | 0.08 | excluded; could not place mutant side chain |
| 3SGB_I_A15R | 2.01 | 1.90 | 0.01 | -- |
| 3SGB_I_A15K | 2.46 | -- | 0.01 | excluded; could not place mutant side chain |
| 3SGB_I_A15E | 0.66 | 2.25 | 0.01 | -- |
| 3SGB_I_A15D | 0.47 | 2.59 | 0.01 | -- |
| 1R0R_I_T17R | 3.30 | 8.00 | 0.03 | -- |
| 1R0R_I_T17K | 1.91 | 3.67 | 0.03 | -- |
| 1R0R_I_T17E | 3.17 | 5.42 | 0.03 | -- |
| 1R0R_I_T17D | 2.85 | 5.63 | 0.03 | -- |
| 1R0R_I_L18R | 2.89 | 6.42 | 0.05 | -- |
| 1R0R_I_L18K | 3.30 | 6.05 | 0.05 | -- |
| 1R0R_I_L18E | 2.15 | 1.49 | 0.05 | -- |
| 1R0R_I_L18D | 4.47 | 4.89 | 0.05 | -- |
| 1R0R_I_E19Y | 1.87 | -- | 0.04 | excluded; could not place mutant side chain |
| 1R0R_I_E19W | 1.83 | -- | 0.04 | excluded; could not place mutant side chain |
| 1R0R_I_E19V | 0.09 | -0.43 | 0.04 | -- |
| 1R0R_I_E19T | 4.20 | 1.97 | 0.04 | -- |
| 1R0R_I_E19S | 3.14 | 2.32 | 0.04 | -- |
| 1R0R_I_E19Q | 1.27 | 0.81 | 0.04 | -- |
| 1R0R_I_E19N | 2.09 | 1.05 | 0.04 | -- |
| 1R0R_I_E19M | 1.55 | 0.63 | 0.04 | -- |
| 1R0R_I_E19L | 0.56 | 1.31 | 0.04 | -- |
| 1R0R_I_E19I | 0.73 | 1.63 | 0.04 | -- |
| 1R0R_I_E19H | 1.69 | -0.79 | 0.04 | -- |
| 1R0R_I_E19G | 2.68 | 1.24 | 0.04 | -- |
| 1R0R_I_E19F | 3.57 | 2.77 | 0.04 | -- |
| 1R0R_I_E19C | 2.34 | 1.95 | 0.04 | -- |
| 1R0R_I_E19A | 2.06 | 1.55 | 0.04 | -- |
| 1R0R_I_A15E | 4.51 | 6.35 | 0.00 | -- |
| 1R0R_I_A15D | 5.15 | 5.66 | 0.00 | -- |
| 1BRS_D_D39A | 7.66 | 10.35 | 0.01 | 100ns; breaking buried salt bridge |
| 1BRS_D_D35A | 4.51 | 2.24 | 0.01 | modeled as protonated, bound and unbound |
| 1BRS_A_R87A | 5.57 | 4.82 | 0.00 | 100ns; breaking buried salt bridge |
| 1BRS_A_R83Q | 5.43 | 8.10 | 0.03 | 100ns; breaking buried salt bridge |
| 1BRS_A_K27A | 5.39 | 5.46 | 0.07 | -- |
| 1BRS_A_H102D | 4.55 | 3.23 | 0.00 | mutant modeled as protonated, bound and unbound |
| 1BRS_A_E73W | 1.66 | -- | 0.01 | excluded; could not place mutant side chain |
| 1BRS_A_E73S | 3.01 | 2.92 | 0.01 | modeled as protonated, bound and unbound |
| 1BRS_A_E73Q | 1.45 | 1.27 | 0.01 | modeled as protonated, bound and unbound |
| 1BRS_A_E73C | 2.53 | 0.96 | 0.01 | modeled as protonated, bound and unbound |
| 1BRS_A_E73A | 2.35 | 0.60 | 0.01 | modeled as protonated, bound and unbound |

Table S2: Summary of results for buried cases

Results using mmGBSA with and without explicit crystallographic water.

| case | experiment (kcal/mol) | Prime mm-GB/SA change in predicted affinity without including crystal waters (kcal/mol) | Prime mm-GB/SA change in predicted affinity without including crystal waters (kcal/mol) |
| --- | --- | --- | --- |
| 1BRS_A_H102D | 4.55 | -1.47 | -1.34 |
| 1BRS_A_R87A | 5.57 | 2.08 | 1.89 |
| 1R0R_I_A15D | 5.15 | -0.48 | -0.34 |
| 1R0R_I_A15E | 4.51 | 0.25 | 0.18 |
| 1BRS_A_E73A | 2.35 | 0.03 | 0.03 |
| 1BRS_A_E73C | 2.53 | 0.02 | 0.02 |
| 1BRS_A_E73Q | 1.45 | 0.04 | 0.03 |
| 1BRS_A_E73S | 3.01 | 0.07 | 0.07 |
| 1BRS_D_D35A | 4.51 | 1.27 | 1.16 |
| 1BRS_D_D39A | 7.66 | 2.08 | 1.89 |
| 3SGB_I_A15D | 0.47 | 1.02 | 1.71 |
| 3SGB_I_A15E | 0.66 | 0.37 | 0.62 |
| 3SGB_I_A15R | 2.01 | -0.31 | -0.51 |
| 3SGB_I_L18D | 5.59 | 0.73 | 1.21 |
| 3SGB_I_L18E | 5.9 | 0.47 | 0.78 |
| 3SGB_I_L18K | 3.14 | 0.86 | 1.44 |
| 3SGB_I_L18R | 3.32 | -0.02 | -0.03 |
| VRC03E91A | 1.51 | 0.56 | 0.8 |
| VRC01E96A | 1.31 | 0.39 | 0.56 |
| 1BRS_A_R83Q | 5.43 | 3.05 | 2.78 |
| 1R0R_I_T17D | 2.85 | 1.71 | 1.22 |
| 1R0R_I_T17E | 3.17 | 1.45 | 1.04 |
| 1R0R_I_T17K | 1.91 | 0.89 | 0.63 |
| 1R0R_I_T17R | 3.3 | 0.53 | 0.38 |
| VRCPG-04E96A | 0.42 | 0.65 | 0.93 |
| 1R0R_I_E19A | 2.06 | 1.31 | 0.94 |
| 1R0R_I_E19C | 2.34 | 0.88 | 0.63 |
| 1R0R_I_E19F | 3.57 | -0.35 | -0.25 |
| 1R0R_I_E19G | 2.68 | 1.66 | 1.19 |
| 1R0R_I_E19H | 1.69 | -0.03 | -0.02 |
| 1R0R_I_E19I | 0.73 | -0.32 | -0.23 |
| 1R0R_I_E19L | 0.56 | -0.33 | -0.23 |
| 1R0R_I_E19M | 1.55 | -0.77 | -0.55 |
| 1R0R_I_E19N | 2.09 | 0.81 | 0.58 |
| 1R0R_I_E19Q | 1.27 | 0.28 | 0.2 |
| 1R0R_I_E19S | 3.14 | 1.4 | 1 |
| 1R0R_I_E19T | 4.2 | 0.35 | 0.25 |
| 1R0R_I_E19V | 0.09 | 0.23 | 0.16 |
| 1R0R_I_L18D | 4.47 | 1.79 | 1.28 |
| 1R0R_I_L18E | 2.15 | 0.82 | 0.59 |
| 1R0R_I_L18K | 3.3 | 2.65 | 1.89 |
| 1R0R_I_L18R | 2.89 | 0.77 | 0.55 |
| 1BRS_A_K27A | 5.39 | 1.74 | 1.59 |
| VRC03D114A | -0.24 | 0.11 | 0.16 |
| 1R0R_I_G32D | 2.85 | 0.18 | 0.13 |
| 1R0R_I_G32E | 1.95 | 0.7 | 0.5 |
| 1R0R_I_G32K | 2.89 | -0.6 | -0.43 |
| 1R0R_I_G32R | 3.42 | -1 | -0.71 |
| VRC01R61A | 0.99 | 1.77 | 2.52 |
| VRC01R71A | 1.64 | 1.03 | 1.47 |
| 1R0R_I_Y20D | 5.69 | 1.68 | 1.2 |
| 1R0R_I_Y20E | 4.55 | 1.16 | 0.83 |
| 1R0R_I_Y20K | 5.43 | 2.43 | 1.73 |
| 1R0R_I_Y20R | 4.41 | 0.62 | 0.45 |
| 3SGB_I_K13A | -2.54 | 0.26 | 0.43 |
| 3SGB_I_K13C | -0.58 | 0.21 | 0.34 |
| 3SGB_I_K13F | -0.72 | 0.21 | 0.35 |
| 3SGB_I_K13H | -0.4 | 0.17 | 0.29 |
| 3SGB_I_K13I | -1.33 | 0 | -0.01 |
| 3SGB_I_K13L | -1.79 | 0.08 | 0.14 |
| 3SGB_I_K13M | -1.15 | -0.09 | -0.15 |
| 3SGB_I_K13N | -0.65 | 0.23 | 0.38 |
| 3SGB_I_K13Q | -0.61 | 0.3 | 0.5 |
| 3SGB_I_K13S | -2.55 | 0.3 | 0.5 |
| 3SGB_I_K13T | -1.92 | 0.2 | 0.33 |
| 3SGB_I_K13V | -0.94 | 0.07 | 0.11 |
| 3SGB_I_T17D | 4.89 | 0.32 | 0.54 |
| 3SGB_I_T17E | 4.73 | 0.13 | 0.78 |
| 3SGB_I_T17K | 2.01 | 0.2 | 0.34 |
| VRC01K52A | 0.62 | 0.64 | 0.92 |
| 1BRS_A_D54A | -0.89 | -0.2 | -0.18 |
| 3SGB_EI19A | 1.02 | 0.34 | 0.56 |
| 3SGB_EI19C | 1.17 | 0.25 | 0.41 |
| 3SGB_EI19F | 1.94 | 0.29 | 0.48 |
| 3SGB_EI19G | 2.1 | 0.61 | 1.01 |
| 3SGB_EI19H | 0.52 | 0.41 | 0.69 |
| 3SGB_EI19I | -0.62 | -0.37 | -0.62 |
| 3SGB_EI19L | 0.78 | -0.11 | -0.19 |
| 3SGB_EI19M | -0.19 | -0.85 | -1.41 |
| 3SGB_EI19N | 1.1 | 0.22 | 0.37 |
| 3SGB_EI19Q | 0.18 | 0 | 0.01 |
| 3SGB_EI19S | 1.94 | 0.3 | 0.5 |
| 3SGB_EI19T | 2.14 | 0.2 | 0.33 |
| 3SGB_EI19V | 0.13 | -0.06 | -0.1 |
| 3SGB_EI19Y | 0.78 | 0.29 | 0.49 |
| 3SGB_I_Y20D | 2.87 | 0.22 | 0.36 |
| 3SGB_I_Y20E | 2.01 | 0.06 | 0.11 |
| 3SGB_I_Y20K | 3.38 | 0.93 | 1.55 |
| 3SGB_I_Y20R | 2.72 | 0.08 | 0.14 |
| VRCPG-04R71A | 2.69 | 1.01 | 1.45 |
| VRC03K52A | 1.11 | 0.68 | 0.97 |
| VRC03R72A | 2.65 | 1.09 | 1.55 |
| 3SGB_I_N36D | 0.9 | -0.01 | -0.02 |
| 3SGB_I_N36E | 0.95 | -0.04 | -0.06 |
| 3SGB_I_N36K | 0.57 | -0.15 | -0.25 |
| 3SGB_I_N36R | 0.57 | -0.56 | -0.94 |
| VRC03R30A | -0.04 | 0.18 | 0.25 |
| VRC03R62A | 1.21 | 1.51 | 2.15 |
| 1BRS_A_R59A | 5.19 | 3.27 | 2.98 |
| 1BRS_A_R59K | 2.49 | 1.96 | 1.79 |
| VRC03D49A | 0.86 | -0.14 | -0.2 |
| 1R0R_I_N36D | 0.56 | 0.08 | 0.05 |
| 1R0R_I_N36E | -0.2 | -0.05 | -0.04 |
| 1R0R_I_N36K | 1.13 | 0.29 | 0.21 |
| 1R0R_I_N36R | 1.44 | 0.6 | 0.43 |
| VRCPG-04R73A | -0.23 | 0.22 | 0.31 |
| 1R0R_I_K13A | -0.6 | -0.06 | -0.05 |
| 1R0R_I_K13C | -0.61 | -0.35 | -0.25 |
| 1R0R_I_K13H | -0.58 | -1.68 | -1.2 |
| 1R0R_I_K13L | -0.32 | -0.99 | -0.71 |
| 1R0R_I_K13M | -0.55 | -1.07 | -0.77 |
| 1R0R_I_K13Q | -0.52 | -0.39 | -0.28 |
| 1R0R_I_K13S | -0.3 | -0.05 | -0.03 |
| 1R0R_I_K13T | 0.88 | 0.21 | 0.15 |
| 1R0R_I_K13V | 0.4 | 0.74 | 0.53 |
| 1R0R_I_K13W | -1.09 | -0.88 | -0.63 |
| 1R0R_I_K13Y | -0.69 | -1.09 | -0.78 |
| VRCPG-04R64A | 1.48 | 0.55 | 0.78 |
| 1BRS_D_E76A | 1.37 | 1.26 | 1.14 |
| VRC01R53A | -0.27 | 1.04 | 1.49 |
| 3SGB_I_R21A | 0.05 | 0.53 | 0.88 |
| 3SGB_I_R21C | 0.54 | 0.52 | 0.87 |
| 3SGB_I_R21F | 0.22 | 0.47 | 0.79 |
| 3SGB_I_R21H | 0.45 | 0.21 | 0.35 |
| 3SGB_I_R21I | 0.24 | 0.32 | 0.53 |
| 3SGB_I_R21L | 0.22 | 0.46 | 0.77 |
| 3SGB_I_R21M | 0.13 | 0.19 | 0.32 |
| 3SGB_I_R21Q | 0.04 | 0.26 | 0.43 |
| 3SGB_I_R21S | 0.27 | 0.51 | 0.85 |
| 3SGB_I_R21T | 0.45 | 0.41 | 0.69 |
| 3SGB_I_R21V | -0.02 | 0.43 | 0.72 |
| 3SGB_I_R21W | 0.31 | 0.54 | 0.9 |
| 3SGB_I_R21Y | 0.29 | 0.47 | 0.78 |
| 1R0R_I_R21A | -0.09 | 0.72 | 0.51 |
| 1R0R_I_R21C | 0.11 | 0.65 | 0.46 |
| 1R0R_I_R21F | 0.44 | 0.75 | 0.53 |
| 1R0R_I_R21I | 0.2 | 0.71 | 0.51 |
| 1R0R_I_R21L | 0.23 | 0.71 | 0.51 |
| 1R0R_I_R21M | 0.44 | 0.21 | 0.15 |
| 1R0R_I_R21N | -0.05 | 0.56 | 0.4 |
| 1R0R_I_R21Q | 0.18 | 0.6 | 0.43 |
| 1R0R_I_R21S | -0.02 | 0.71 | 0.5 |
| 1R0R_I_R21T | 0.34 | 0.66 | 0.47 |
| 1R0R_I_R21V | 0.11 | 0.7 | 0.5 |
| 1R0R_I_R21W | 0.93 | 0.82 | 0.59 |
| 1R0R_I_R21Y | 0.81 | 0.74 | 0.53 |
| VRC01D99A | 0.05 | 0.02 | 0.03 |
| 1BRS_D_E80A | 0.54 | 0.03 | 0.02 |
| VRCPG-04D74A | -0.74 | 0.06 | 0.08 |
| VRC03D110A | -0.38 | 0.01 | 0.01 |

Table S3: Comparison of mm-GB/SA including crystallographic waters vs mm-GB/SA without

Lambda Weights

The non-uniform lambda weights used for the electrostatic term of the energy in intermediate lambda windows are as follows from the wild or mutant end state to the mutant or wild end:

| window | wild type electrostatic weight |
| --- | --- |
| 0 | 1.000 |
| 1 | 0.996 |
| 2 | 0.984 |
| 3 | 0.965 |
| 4 | 0.938 |
| 5 | 0.902 |
| 6 | 0.859 |
| 7 | 0.809 |
| 8 | 0.750 |
| 9 | 0.684 |
| 10 | 0.609 |
| 11 | 0.527 |
| 12 | 0.438 |
| 13 | 0.340 |
| 14 | 0.234 |
| 15 | 0.121 |
| 16 | 0.000 |
| 17 | 0.000 |
| 18 | 0.000 |
| 19 | 0.000 |
| 20 | 0.000 |
| 21 | 0.000 |
| 22 | 0.000 |
| 23 | 0.000 |

Table S4: Non-uniform electrostatic weights used in FEP lambda schedule. The weights are used symmetrically; the mutant type weights start from 23 and run to 0.
